# Predictive Model for Estimating Annual *Ebolavirus* Spillover Potential 

**DOI:** 10.3201/eid3104.241193

**Published:** 2025-04

**Authors:** Carson T. Telford, Brian R. Amman, Jonathan S. Towner, Joel M. Montgomery, Justin Lessler, Trevor Shoemaker

**Affiliations:** Centers for Disease Control and Prevention, Atlanta, Georgia, USA (C.T. Telford, B.R. Amman, J.S. Towner, J.M. Montgomery, T. Shoemaker); University of North Carolina, Chapel Hill, North Carolina, USA (C.T. Telford, J. Lessler); Johns Hopkins University, Baltimore, Maryland, USA (J. Lessler)

**Keywords:** Ebolavirus, viruses, zoonoses, zoonotic spillover, prediction, forest change, modeling

## Abstract

Forest changes, human population dynamics, and meteorologic conditions have been associated with zoonotic *Ebolavirus* spillover into humans. High-resolution spatial data for those variables can be used to produce estimates of spillover potential and assess possible annual changes. We developed a model of *Ebolavirus* spillover during 2001–2021, accounting for variables measured across multiple spatial and temporal scales. We estimated the annual relative odds of *Ebolavirus* spillover during 2021 and 2022. The highest relative spillover odds estimates occurred in patches that closely followed the spatial distribution of forest loss and fragmentation. Regions throughout equatorial Africa had increased spillover estimates related to changes in forests and human populations. Spillover events in 2022 occurred in locations in the top 0.1% of overall spillover odds estimates or where estimates increased from 2021 to 2022. This model can be used to preemptively target surveillance to identify outbreaks, mitigate disease spread, and educate the public on risk factors for infection.

Zoonotic spillover of *Ebolavirus* species into humans was identified approximately 1 time/year during 2000–2022 (*1*). Although the first documented Ebola virus disease (EVD) outbreak occurred almost 50 years ago, the determinants of *Ebolavirus* spillover (transmission from an animal reservoir to a human) remain poorly understood (*1*,*2*). *Ebolavirus* species known to cause human infection include *Zaire ebolavirus* (ZEBOV), *Sudan ebolavirus* (SUDV), *Bundibugyo ebolavirus* (BDBV), and *Tai Forest ebolavirus* (TAFV). A natural reservoir has not been confirmed for any of those species, but forest-dwelling bats have been suggested as likely candidates, and each species might have a unique reservoir species (*3**–**6*). Investigations of outbreaks (>1 case) have linked *Ebolavirus* spillover into humans with bushmeat hunting activities, handling bushmeat in market settings, and general proximity and possible contact with bats (*7*).

Studies of *Ebolavirus* spillover ecology have found forest density and meteorologic variability as potential predictors of the viruses in the environment and possible spillover into humans (*5*,*8*–*10*). Forest loss and fragmentation (division of forests into patches) have also been associated with spillover (*11*,*12*). However, the mechanism behind that association is unclear. Research of zoonotic spillover of Hendra virus from bats in Australia has found that disruption of natural habitats can lead to elevated immunologic stress in bats, leading to increased viral shedding (*13*). A lack of native food sources then results in bats foraging less nutritious resources closer to susceptible humans and horses, leading to spillover (*13*,*14*). Such processes could reasonably occur within the ecologic cycle of *Ebolavirus* because natural forests continue to be altered throughout equatorial Africa. However, research on host behavioral changes and *Ebolavirus*-specific ecologic dynamics are unlikely to be productive until a natural reservoir is identified.

Notwithstanding those limitations, public health workers are tasked to identify and respond to EVD outbreaks. Leveraging existing and available knowledge and tools could be valuable to refining public health risk mitigation strategies to target surveillance and public education campaigns toward specific locations and times when the potential for spillover is highest. 

High-resolution data on forest cover, forest changes, and human population distribution are available annually, typically several months after a year has ended, providing an opportunity to generate annual updated estimates of *Ebolavirus* spillover potential on the basis of evolving ecologic contexts (*15*,*16*). We aimed to develop a predictive model to generate annual estimates of *Ebolavirus* spillover likelihood, accounting for annual changes to forests and human populations; to identify changes in spillover likelihood and to apply predictions to 2022 spillover events identified in that year.

## Methods

### *Ebolavirus* Spillover Data

We represented spillover events as geographic coordinates for the village of residence of human EVD outbreak index cases from 2001–2022 that were not linked to latently infected survivors from past outbreaks (*17*). We sourced coordinates from published literature and outbreak reports. Outbreaks without reported coordinates or village names were confirmed through consultation with outbreak responders from the US Centers for Disease Control and Prevention. We began our analysis in 2001 because high-resolution forest loss and human population data became available that year (*15*,*16*). We identified 24 isolated spillover events during 2001–2022: 17 ZEBOV, 5 SUDV, and 2 BDBV (Appendix Table 1). Because spillover of TAFV was only identified in 1994, we excluded it from this analysis.

### Spillover Predictors

**Figure 1:**
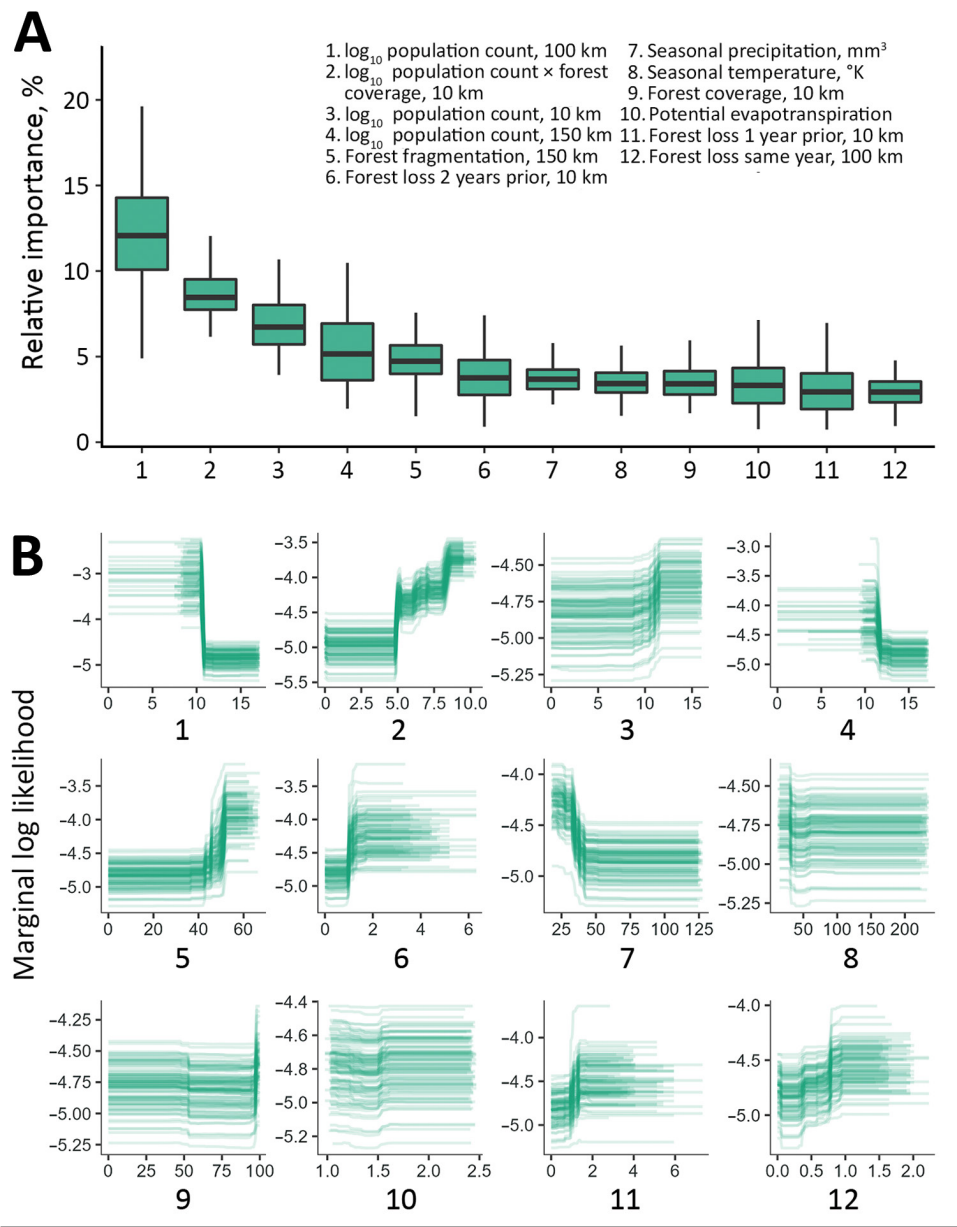
Relative effects of variables in a multispecies predictive model for estimating annual *Ebolavirus* spillover potential. A) Overall variable effects for predicting *Ebolavirus* spillover likelihood; B) marginal response curves of the relationship between spillover likelihood and each covariate for the top 12 predictors across ensemble of 100 models. Graphs show effects for the multispecies *Ebolavirus* inputs combining the species *Zaire ebolavirus*, *Sudan ebolavirus*, *Bundibugyo ebolavirus*, and *Tai Forest ebolavirus*. Boxes indicate interquartile ranges, horizontal lines within boxes indicate medians, whiskers indicate range values. We measured forest loss in individual covariates for the same year, 1 year prior, and 2 years before spillover events. Mean values for percentage of forest cover, forest loss, forest fragmentation, and human population were extracted surrounding each spillover location using mutually exclusive circular donut buffers with maximum radii of 10, 25, 50, 100, and 150 km.

We ascertained predictors of spillover within 10 degrees latitude of the Equator in Africa (Appendix Figure 1). We only included locations with a historical average >500 mm of annual precipitation because spillover historically has occurred in vegetation-dense areas, and our goal was to leverage a model to elucidate patterns to predict spillover risk among regions where ebolaviruses have historically been identified (*10*). We calculated annual forest cover percentage and loss at a spatial resolution of 1 km^2^ (*15*). We analyzed forest fragmentation by first defining forests as locations with >70% cover, then we classified fragmented areas according to previously described methods that considered patch, transitional, edge, and perforated forest areas to be fragmented (*18*). We conducted a sensitivity analysis using 80% as the cutoff to define forests. We log transformed annual human population count data because of skewed distributions in population centers that could have excessive influence on predictions (*16*). We created a product term combining human population count and forest cover to represent high density of both susceptible persons and vegetation. We analyzed additional covariates as historical annual averages, including potential evapotranspiration (PET), night-time land surface temperature (NTLST), elevation, temperature seasonality (SD of monthly temperature), and precipitation seasonality (coefficient of variation of monthly precipitation) (Appendix Table 2, Figure 1). We opted to exclude covariates related to the distribution of putative *Ebolavirus* reservoirs because composite distributions of suspect reservoirs are highly correlated with forest cover, which we already included in the model (*8*).

### Spatial and Temporal Scales of Covariate Measurement

The temporal lag between habitat changes and *Ebolavirus* spillover risk is unknown, so we measured forest loss in different covariates for the same year, 1 year prior, and 2 years before spillover events. We extracted mean values for percentage of forest cover, forest loss, forest fragmentation, and human population surrounding each spillover location using mutually exclusive circular donut buffers with maximum radii of 10, 25, 50, 100, and 150 km. We used multiple spatial measurement scales because the scale at which habitat changes are related to behavior of natural reservoirs, humans, and other susceptible animal species is unclear. Elevation, PET, NTLST, temperature seasonality, precipitation seasonality, and the term combining human population and forest cover were analyzed as average values within 10 km of each spillover location. The model included 36 total covariates, 30 scaled and 6 unscaled.

### Analysis and Prediction

We compared conditions surrounding spillover events against 10,000 randomly generated absence locations within the study area, which we randomly assigned to a year from 2001 to 2021, representing a time and location at which spillover was not identified (Appendix Figure 2). We weighted absence locations on the basis of log population count to account for increased reporting in populated areas. In the sensitivity analysis, we used coordinates for known health centers as absence locations (*19*).

We used boosted regression trees to fit binomial models estimating the odds of spillover for events that occurred during 2001–2021 (*20*) in R (The R Project for Statistical Computing, https://www.r-project.org). Trees-based machine learning algorithms are robust for analyzing many covariates because they analyze subsets of covariates, reducing bias when correlation exists between covariates, which was the case for our analysis of multiple spatial and temporal scales. We performed 2 separate analyses: 1 that included spillover of ZEBOV, BUBV, and SUDV (termed the multispecies analysis), and an ZEBOV-only analysis because most spillover events resulted from ZEBOV. Each analysis fit an ensemble of 100 models to prevent overfitting, sampling 50 absence locations per presence location in each model. We used area under the receiver operator curve (AUC) and a leave-1-year-out cross validation to evaluate the predictive ability of each model ensemble. We determined sensitivity and specificity by using a fitted odds cutoff that maximized the product. We reported relative predictive importance and marginal response curves for the top 12 predictors. We fit models to covariate values on a grid of the study area to predict the odds of spillover in 2021 and 2022. For each prediction grid location, we reported the relative odds ratios (RORs), defined as the fitted spillover odds divided by the mean spillover odds across the entire study area each year. We emphasized describing the top 1% of prediction locations. We assessed the predicted RORs for 2022 and 1-year changes in RORs from 2021 to 2022 at the locations with 2 spillover events in that year that were not included in model training. Then, we repeated both analyses, excluding covariates related to forest loss and fragmentation to assess the contribution of those variables to predictions.

## Results

### Model Fit

**Figure 2:**
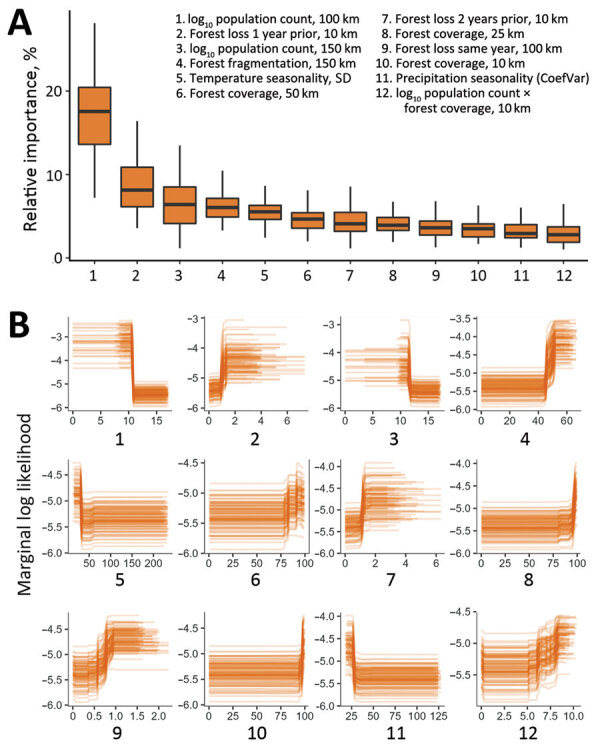
Relative effects of variables in a *Zaire ebolavirus*–only predictive model for estimating annual *Ebolavirus* spillover potential. A) Overall variable effects for predicting *Ebolavirus* spillover likelihood; B) marginal response curves of the relationship between spillover likelihood and each covariate for the top 12 predictors across ensembles of 100 models. Boxes indicate interquartile ranges, horizontal lines within boxes indicate medians, whiskers indicate range values. We measured forest loss in individual covariates for the same year, 1 year prior, and 2 years before spillover events. Mean values for percentage of forest cover, forest loss, forest fragmentation, and human population were extracted surrounding each spillover location using mutually exclusive circular donut buffers with maximum radii of 10, 25, 50, 100, and 150 km.

The cross-validation AUC was 0.88 for the multispecies analysis and was 0.92 for the ZEBOV-only analysis. In the multispecies analysis classification, sensitivity was 86.4% and specificity was 72.7%; in the ZEBOV-only analysis, sensitivity was 93.8% and specificity was 86.6% (Appendix Figure 3). In both analyses, human population and forest-related variables were among the top predictors at multiple spatial and temporal measurement scales. Marginal response curves for human population showed that, at the 50–100-km scale, spillover likelihood decreased as human population increased; however, at a smaller scale, within 10 km, likelihood increased as human population increased. Forest loss and fragmentation both had positive relationships with spillover likelihood across spatial and temporal measurement scales. Average precipitation seasonality was also among the top predictors, and spillover likelihood was highest where precipitation was more stable. Relative importance and marginal response curves were similar between the multispecies and ZEBOV-only analyses, although lower human population sizes at a larger spatial scale, forest loss, and forest fragmentation had greater predictive weight in the ZEBOV-only analysis (Figures 1, 2).

### Relative Spillover Odds Estimates for 2022

**Figure 3:**
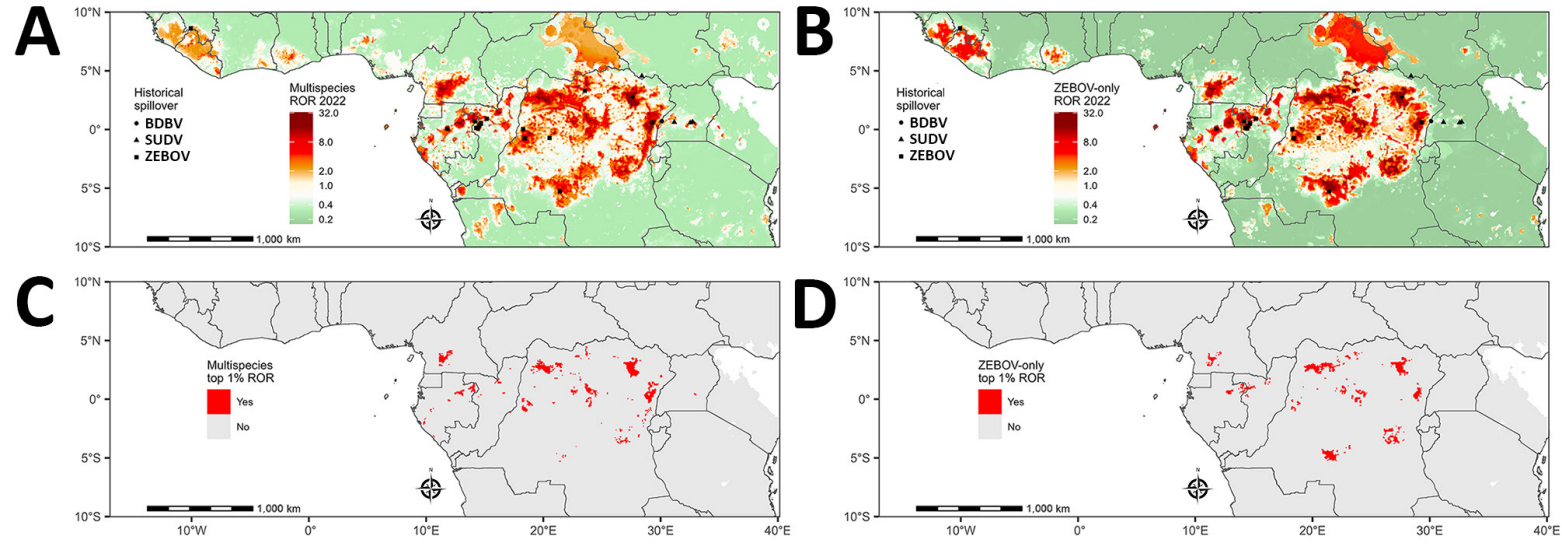
RORs for spillover from predictive model for estimating annual *Ebolavirus* spillover potential. A, B) RORs for spillover in 2022 in the multispecies (A) and ZEBOV-only (B) models. C, D) Top 1% RORs (1-km^2^ grid cells) in multispecies (C) and ZEBOV-only (D) models. Multispecies model combined BDBV, SUDV, and ZEBOV. RORs were calculated by using the estimated odds of *Ebolavirus* spillover divided by the average estimate across the entire study area. BDBV, *Bundibugyo ebolavirus*; ROR, relative odds ratio; SUDV, *Sudan ebolavirus*; ZEBOV, *Zaire ebolavirus*.

In the multispecies analysis, estimated RORs in 2022 ranged from 0.3 to 32.3 (Figure 3). The top percentile of ROR predictions was among those that exceeded 10.1, which occurred in 7 countries: Democratic Republic of the Congo (DRC; maximum 32.3), Republic of the Congo (ROC; maximum 28.1), Gabon (maximum 25.9), Cameroon (maximum 20.8), Uganda (maximum 13.2), Equatorial Guinea (maximum 12.5), and Central African Republic (maximum 10.2). Among prediction locations in the top percentile, 79.1% were in DRC, 9.1% in Cameroon, 8.6% in Gabon, and 2.5% in ROC; <1% occurred in each Uganda, Central African Republic, and Equatorial Guinea.

In the ZEBOV-only analysis, RORs in 2022 ranged from 0.2 to 31.2 (Figure 3). The top percentile of ROR predictions was among those that exceeded 12.5, which occurred in 4 countries: DRC (maximum 31.2), Gabon (maximum 23.6), ROC (maximum 23.6), and Cameroon (maximum 23.4). Among prediction locations in the top percentile, 83.4% occurred in DRC, 6.5% in Cameroon, 6.1% in Gabon, and 4.1% in ROC (Figure 3). The spatial distributions of predictions were similar between the analyses, but those from the ZEBOV-only analysis tended to be higher in regions of dense forest, whereas those from the multispecies analysis tended to be higher along forest edges (Appendix Figure 4).

### Changes in RORs from 2021–2022

**Figure 4:**
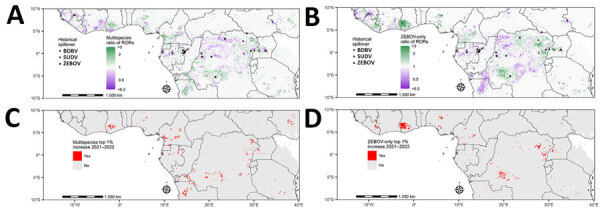
Change in estimated spillover RORs in 2022 compared with 2021 from a predictive model for estimating annual *Ebolavirus* spillover potential. A, B) Ratio of RORs for spillover in 2022 relative to RORs in 2021 in the multispecies (A) and ZEBOV-only (B) models. C, D) Top 1% ratio of RORs in multispecies (C) and ZEBOV-only (D) models, highlighting top 1% of prediction cells with 1-year increases in spillover ROR estimates from 2021 to 2022. Multispecies model combined BDBV, SUDV, and ZEBOV. RORs were calculated by using the estimated odds of *Ebolavirus* spillover divided by the average estimate across the entire study area. BDBV, *Bundibugyo ebolavirus*; ROR, relative odds ratio; SUDV, *Sudan ebolavirus*; ZEBOV, *Zaire ebolavirus*.

Spillover ROR estimates in 2022 increased in 24.9% of the study area compared to estimates in 2021, due to changes to forests and human population size. The ratio between ROR estimates in 2022 relative to estimates in 2021 (1-year increase) ranged from 0.1 to 4.2, indicating that spillover potential both increased and decreased across the study area (Figure 4). The top percentile of 1-year increases in ROR estimates was those that increased by >1.8 times, which occurred in 19 countries. The countries that had the most prediction locations in the top percentile of 1-year ROR estimate increases were DRC (36.3%), Cameroon (15.6%), and Angola (11.7%).

In the ZEBOV-only analysis, ROR estimates in 2022 were higher than estimates in 2021 in 18.4% of the study area. The ratio between ROR estimates in 2022 compared to estimates in 2021 (1-year increase) ranged from 0.1 to 5.9 (Figure 4). The top percentile of 1-year increases in RORs was among those exceeding 1.9 times RORs from the previous year, which occurred in 17 countries. Countries with the most prediction locations in the top percentile of 1-year ROR increases were DRC (37.9%), Ghana (27.3%), and Nigeria (8.7%).

### Estimated Spillover Odds at 2022 Spillover Sites

**Figure 5:**
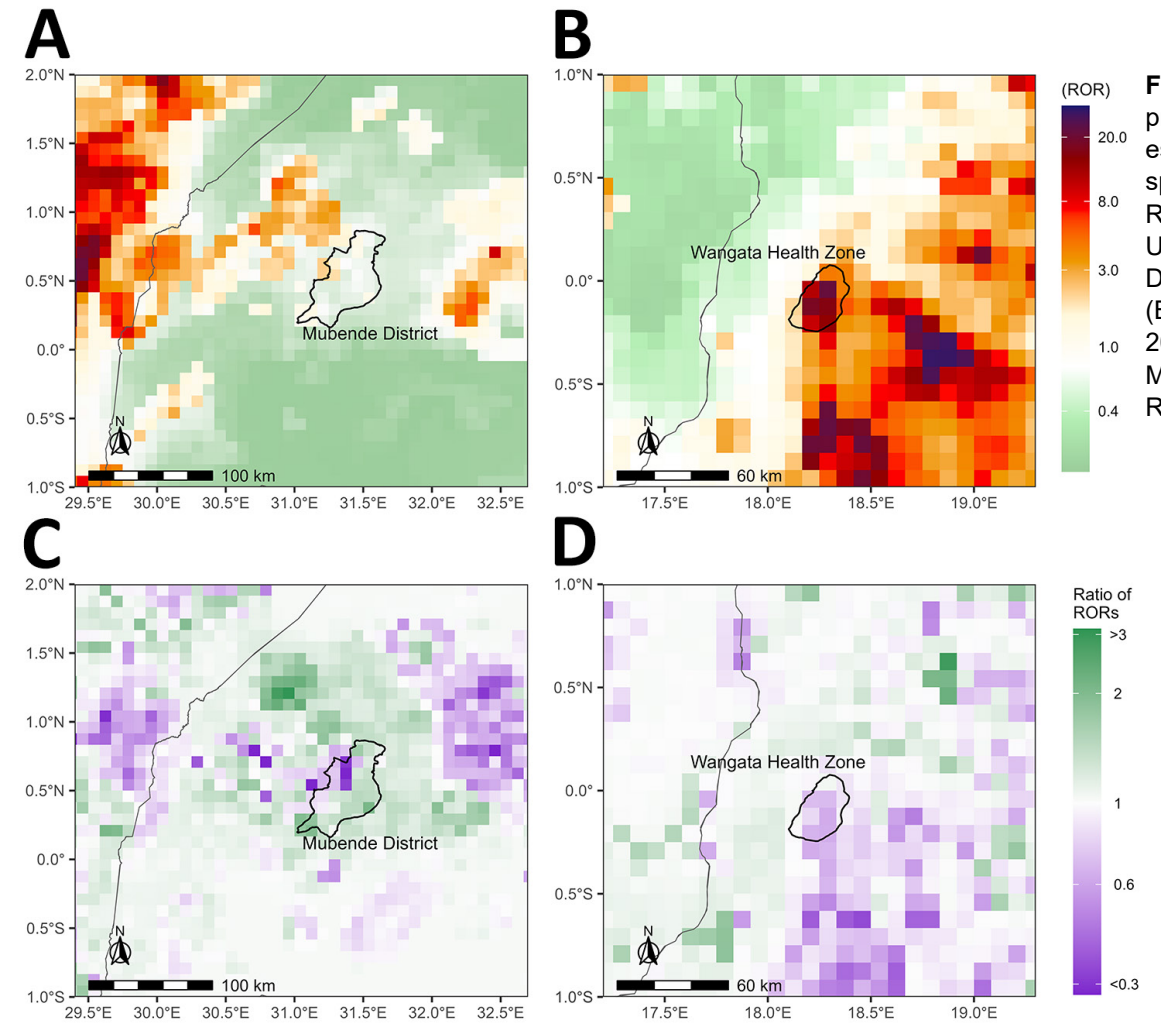
Heatmaps of ROR predictions from a model for estimating annual *Ebolavirus* spillover potential. A, B) Estimated RORs for spillover in Mubende, Uganda (A), and Mbadanka, Democratic Republic of the Congo (B). C, D) Ratios of RORs from 2022 relative to those from 2021 for Mubende (C) and Mbadanka (D). ROR, relative odds ratio.

Two *Ebolavirus* spillover events were identified in 2022, and we did not use those in model training. Spillover of ZEBOV was identified in the Wangata Health Zone in Mbandaka, DRC. The maximum spillover ROR estimate from the ZEBOV-only analysis in Wangata was 21.5, which ranked within the top 0.1 percentile in Equatorial Africa and, for DRC specifically, ranked in the top 0.3 percentile. Wangata Health Zone was also the location of the single highest overall 1-km^2^ prediction cell in Equatorial Africa in 2021 (ROR 31.3). The second spillover in 2022 resulted from SUDV in the Mubende district of Uganda, which had a maximum estimated spillover ROR of 2.0 from the multispecies analysis, ranking in the top 12 percentile of spillover ROR estimates in 2022 and the top 6 percentile in Uganda. During 2021–2022, a large proportion of Mubende district also saw an increase in estimated spillover RORs from the multispecies analysis; the ROR prediction in 2022 increased by <2.1 times that in 2021, which ranked in the top 0.3 percentile in both Equatorial Africa and Uganda (Figure 5).

Sensitivity analysis using health facility coordinates as absence locations produced spatial distributions of ROR predictions similar to those of the primary analysis, ranging from 0.4 to 33.5 in the multispecies analysis and from 0.2 to 54.9 in the ZEBOV-only analysis (Appendix Figure 5). For the multispecies analysis, the correlation coefficient between predictions in the primary analysis and the sensitivity analysis using health facilities as absence locations was 0.71 and for ZEBOV-only analyses was 0.69 (Appendix Figure 6). The sensitivity analysis classifying fragmented forests based on a forest cover definition of >80% forest cover (compared with 70% cover) also produced spatial trends in RORs similar to those in the primary analysis but resulted in spillover ROR estimates up to 5 times as high in western DRC and southern Cameroon in the ZEBOV-only analysis (Appendix Figure 7).

### Difference between Full and Reduced Models

Compared with reduced models that ignored forest loss and fragmentation covariates, full models that accounted for those covariates produced considerable differences in the spatial pattern of estimated spillover RORs across Equatorial Africa. The highest estimates of spillover odds in the reduced version of the ZEBOV-only analysis were concentrated in a band along the Equator, primarily in the center of the rainforest in DRC, ROC, and Gabon. Models accounting for forest changes highlighted patches of elevated estimates away from the center of the rainforest, such as in the forest edges in DRC, southern Cameroon, and coastal West Africa (Appendix Figure 8). Of note, we identified West Africa as a region with spillover RORs >1 only after accounting for forest change covariates.

## Discussion

The primary goal of this analysis was to develop a model to generate annually updated estimates of the relative odds of *Ebolavirus* spillover, accounting for changes to forests, human populations, and meteorologic conditions. We highlighted 1-year changes in spillover odds resulting from recent changes in environmental and population conditions. Prediction locations that had the highest spillover odds estimates were in patches throughout Equatorial forests, and forest loss and fragmentation had considerable impact on the spatial distribution of predictions. Patches with the highest predictions of spillover odds were mostly in DRC, Cameroon, Gabon, and ROC. Prediction locations with the largest 1-year increases in spillover odds from 2021 to 2022 were more widespread, the largest patches occurred in southern Ghana, Nigeria, Cameroon, Gabon, northern Angola, Uganda, Ethiopia, and near forest borders throughout DRC. Assessment of predictions at the locations of 2 spillover events that occurred in 2022 in DRC and Uganda found that the spillover of ZEBOV in DRC occurred in a location that ranked in the top percentile of overall spillover odds predictions, and the spillover of SUDV in Uganda occurred in a location ranking in the top percentile of 1-year increases in predictions for 2022 compared with 2021.

This predictive modeling effort can address questions around which public health actions can and should be taken as a result of spillover odds predictions. For a rare event, such as *Ebolavirus* spillover, conducting active surveillance in all regions with above-average estimates of spillover likelihood or 1-year increases in spillover potential, is unlikely to be a good investment. However, spillover odds estimates or changes in estimates could be used as a tool for public health programs and surveillance efforts to prioritize locations with the highest predicted potential for spillover. In locations with the highest estimated spillover RORs, or largest 1-year increases, public health programs may focus on training local healthcare providers on case identification, especially among high-risk groups, such as hunters or miners, and subsequent reporting of suspect infections through a symptom-based reporting infrastructure. Targeting those strategies toward the locations ranking in top 0.3% of RORs or 1-year increases in ROR in our analysis would have correctly allocated efforts in locations where spillover was identified in 2022. *Ebolavirus* spillover has historically occurred in resource-limited regions that may have limited communication with public health surveillance networks. Such efforts would not only build capacity of local healthcare providers but also open lines of communication between public health programs and local health providers. Improved communication and routine symptom-based reporting in locations with high predicted spillover potential are low-cost goals that would benefit public health infrastructure beyond just *Ebolavirus* surveillance (*21*,*22*).

Aside from targeted surveillance and healthcare provider training, consideration should also be given to the underlying ecologic drivers of spillover. Bushmeat is a vital protein source for many persons in rural regions throughout the world. Although bushmeat has been associated with *Ebolavirus* spillover, total prevention of bushmeat consumption is likely not an attainable goal. However, bushmeat trade is heavily intertwined with logging activities, which have resulted in unsustainable growth of bushmeat markets and increased contact between humans and wildlife (*23*,*24*). Strategies to mitigate the harmful effects of bushmeat hunting should also account for its role in different cultural contexts and its necessity for populations lacking other food sources. Another study proposed solutions such as preventing or taxing transportation of bushmeat to prevent commercial bushmeat sales, providing small livestock production to substitute bushmeat consumption, and requiring logging companies to fund employment of independent conservation officers (*25*). Protecting forests can also mitigate the risk for spillover, because a wider ecosystem for bats can dilute congregations of bats at sites with limited resources. In contrast, deforestation is often followed by agricultural activities, such as fruit crop cultivation, which can draw bats into proximity with humans and increase potential for spillover.

Estimates of annual shifts in spillover odds may be valuable for considering where and when targeted sampling could be done to study potential *Ebolavirus* reservoirs. In areas undergoing forest loss, studies could evaluate changes in animal behavior and seroprevalence among suspect reservoirs and human populations, especially if environmental changes correlate with bat birthing patterns, which have been shown to be correlated with increased filovirus circulation among bat populations (*26*,*27*). 

Differences in results between the multispecies analysis and the ZEBOV-only analysis lend support to previous observations of the distinct ecologic context surrounding different types of Ebolaviruses. When limiting analysis to ZEBOV, forest loss and fragmentation and low population density at large spatial scales were more relevant to prediction accuracy compared with the multispecies analysis, and corresponding predictions were higher in regions of denser forest. In contrast, including SUDV and BDBV spillover in the multispecies analysis, predictions were lower in the dense forest and higher in forest border regions. One-year changes in spillover odds estimates also varied between the 2 analyses. According to the ZEBOV-only analysis, spillover RORs increased in eastern Liberia and decreased in northern Angola in 2022, whereas the multispecies analysis predicted decreased spillover RORs in eastern Liberia and increased in northern Angola. Given that the only difference between the multispecies and ZEBOV-only analyses was the inclusion of SUDV and BDBV spillover events, we hypothesize that unique environmental conditions represented in this model may be associated with emergence of each *Ebolavirus* species.

The models used to generate the estimates we report were predictive models, for which interpretations of model output are distinct from etiologic or causal models. Causal interpretation of any exposure and outcome relationship should be done with rigorous consideration of exposure and outcome relationships, controlling for known confounding variables, and not controlling for colliders on the causal pathway (*28*). In contrast, predictive models prioritize accurate predictions over interpretation of covariate coefficients. As such, many covariates may be included without necessary consideration for why it results in accurate predictions. For example, why forest change variables at certain spatial scales of measurement were among the top predictors of *Ebolavirus* spillover was unclear; those variables may only be correlated with or act as proxies for unmeasured variables that are true causes of the outcome. Thus, interpretation of results related to the underlying causes of spillover should be done with caution. Our predictions represent locations where an index case of an outbreak is likely to be identified and not necessarily the location where spillover occurred. Incorporating multiple spatial scales surrounding the village of an index case are meant to encompass areas where infection may have occurred and surrounding context leading to the spillover event. 

Although we found forest fragmentation to be a primary predictor of spillover, its relationship with spillover could change depending on how fragmentation is defined. The method we used requires a binary definition of forests before determining which areas surrounding forests would be considered fragmented (*18*)*.* We found that spatial trends remained similar between 2 different percentages of forest cover cutoffs, but ROR estimates were higher in the center of the rainforest when using an 80% cutoff and higher along forests edges when using a 70% cutoff (Appendix Figure 7). Using lower cutoffs to define forests when classifying fragmentation would expand the area of fragmented forests and possibly identify additional areas of lower forest cover with potential for spillover.

In summary, zoonotic *Ebolavirus* spillover poses a major threat to human and animal health in Equatorial Africa. Although the underlying ecologic process of spillover remains poorly understood, spillover is positively correlated with forest loss and fragmentation and differentially affected by human population size, depending on the spatial scale of analysis. High-resolution data on forest cover and human population distributions are publicly available and annually updated. Leveraging those data to estimate the absolute and relative changes in spillover potential can provide valuable information to help public health officials prioritize surveillance and communication with populations living and serving in regions that are at high risk for EVD emergence.

Appendix Additional information on a predictive model for estimating annual Ebolavirus spillover potential.
