# Supplementary material for: Predictive Model for Estimating Annual Ebolavirus Spillover Potential
Source: Emerg Infect Dis. 2025 Apr;31(4):689–98. doi: 10.3201/eid3104.241193 (PMC11950271; doi:10.3201/eid3104.241193)
Supplement: Appendix — Additional information on a predictive model for estimating annual Ebolavirus spillover potential. [file 24-1193-Techapp-s1.pdf]

Article DOI: https://doi.org/10.3201/eid3104.241193

EID cannot ensure accessibility for supplementary materials supplied by authors. Readers who have difficulty accessing supplementary content should contact the authors for assistance.

Predictive Model for Estimating Annual *Ebolavirus* Spillover Potential

Appendix

Appendix Table 1. Spillover events used in a predictive model for estimating annual *Ebolavirus* spillover potential*

ID	Country	*Ebolavirus* species	Year	Lat	Long	Source
1	Gabon	*Zaire*	2001	0.0550	11.8109	(*1*), (*2*)
2	Gabon	*Zaire*	2001	0.7001	14.1580	(*3*), (*4*), (*5*)
3	ROC	*Zaire*	2002	0.1333	14.2667	(*2*), (*5*)
4	ROC	*Zaire*	2002	0.2987	14.5075	(*6*)
5	ROC	*Zaire*	2003	0.0682	14.4200	(*2*), (*5*), (*7*)
6	ROC	*Zaire*	2003	0.5619	14.6573	(*5*)
7	South Sudan	*Sudan*	2004	4.5568	28.4016	(*8*)
8	ROC	*Zaire*	2004	0.9064	15.1751	(*9*)
9	ROC	*Zaire*	2005	0.4944	14.6786	Trevor Shoemaker, pers. comm., 2022 Jun 6
10	DRC	*Zaire*	2007	−5.259	21.4095	(*2*)
11	Uganda	*Bundibugyo*	2007	0.7038	30.1175	(*7*), (*10*), (*11*)
12	DRC	*Zaire*	2008	−5.24	21.4103	(*7*), (*12*)
13	Uganda	*Sudan*	2011	0.6444	32.7276	(*7*), (*13*), (*14*)
14	Uganda	*Sudan*	2012	0.6214	31.1685	(*7*), (*12*)
15	DRC	*Bundibugyo*	2012	2.7718	27.6196	(*7*)
16	Uganda	*Sudan*	2012	0.5784	32.5480	(*11*)
17	Guinea	*Zaire*	2013	8.6225	−10.064	(*7*)
18	DRC	*Zaire*	2014	−0.714	20.5302	(*15*)
19	DRC	*Zaire*	2017	3.2990	23.5430	(*16*), (*17*)
20	DRC	*Zaire*	2018	−0.737	18.4214	(*18*); Trevor Shoemaker, pers. comm., 2022 Jun 6
21	DRC	*Zaire*	2018	0.6059	29.3065	Trevor Shoemaker, pers. comm., 2022 Jun 6
22	DRC	*Zaire*	2020	0.0300	18.2800	Trevor Shoemaker, pers. comm., 2022 Jun 6
23	DRC	*Zaire*	2022	0.0414	18.2770	Trevor Shoemaker, pers. comm., 2022 Jun 6
24	Uganda	*Sudan*	2022	0.6697	31.4686	Trevor Shoemaker, pers. comm., 2022 Jun 6

*CDC, Centers for Disease Control and Prevention; DRC, Democratic Republic of the Congo; ROC, Republic of the Congo.

Appendix Table 2. Variable descriptions for a predictive model for estimating annual *Ebolavirus* spillover potential

Variable	Units	Original spatial resolution	Years represented in data	Temporal range of analysis	Citation
Elevation	Meters	1×1 km	2000	Single time point	(*19*)
Forest cover (FC)	Percentage	30×30 m	2000–2022	% FC during year of spillover	(*20*)
Forest fragmentation	Binary	30×30 (calculated using FC data)	2000–2022	% Fragmentation during year of spillover	(*21*)
Forest loss	Binary	30×30 m	2001–2022	% forest loss during same year, 1 y prior, and 2 years before spillover	(*20*)

Variable	Units	Original spatial resolution	Years represented in data	Temporal range of analysis	Citation
Night-time land surface temperature (NTLST)	Degrees Kelvin	0.05 degrees	2000–2022	Time Averaged mean monthly values	(*22*)
Potential evapotranspiration (PET)	mm day-1	1×1 km	2000–2022	Time Averaged mean monthly values	(*23*)
Precipitation	Millimeters	1×1 km	1970–2000	Time Averaged mean monthly values	(*19*)
Temperature seasonality	Standard Deviation	1×1 km	1970–2000	Time Averaged mean monthly values	(*19*)
Precipitation seasonality	Coefficient of Variation	1×1 km	1970–2000	Time Averaged mean monthly values	(*19*)
Population count	Population per grid cell	30 arc seconds (≈1× km)	2000–2022	Population per grid cell during year of spillover	(*24*)

References

1. Lahm SA, Kombila M, Swanepoel R, Barnes RF. Morbidity and mortality of wild animals in relation to outbreaks of Ebola haemorrhagic fever in Gabon, 1994–2003. Trans R Soc Trop Med Hyg. 2007;101:64–78. PubMed https://doi.org/10.1016/j.trstmh.2006.07.002

2. Nkoghe D, Kone ML, Yada A, Leroy E. A limited outbreak of Ebola haemorrhagic fever in Etoumbi, Republic of Congo, 2005. Trans R Soc Trop Med Hyg. 2011;105:466–72. PubMed https://doi.org/10.1016/j.trstmh.2011.04.011

3. Leroy EM, Souquière S, Rouquet P, Drevet D. Re-emergence of Ebola haemorrhagic fever in Gabon. Lancet. 2002;359:712. PubMed https://doi.org/10.1016/s0140-6736(02)07796-6

4. Allela L, Boury O, Pouillot R, Délicat A, Yaba P, Kumulungui B, et al. Ebola virus antibody prevalence in dogs and human risk. Emerg Infect Dis. 2005;11:385–90. PubMed https://doi.org/10.3201/eid1103.040981

5. Pourrut X, Kumulungui B, Wittmann T, Moussavou G, Délicat A, Yaba P, et al. The natural history of Ebola virus in Africa. Microbes Infect. 2005;7:1005–14. PubMed https://doi.org/10.1016/j.micinf.2005.04.006

6. Rouquet P, Froment JM, Bermejo M, Kilbourn A, Karesh W, Reed P, et al. Wild animal mortality monitoring and human Ebola outbreaks, Gabon and Republic of Congo, 2001–2003. Emerg Infect Dis. 2005;11:283–90. PubMed https://doi.org/10.3201/eid1102.040533

7. Mylne A, Brady OJ, Huang Z, Pigott DM, Golding N, Kraemer MUG, et al. A comprehensive database of the geographic spread of past human Ebola outbreaks. Sci Data. 2014;1:140042. PubMed https://doi.org/10.1038/sdata.2014.42

8. World Health Organization. Institutional repository for informational sharing: Ebola [cited 2022 June 6]. https://iris.who.int/discover?query=ebola&filtertype=subject&filter_relational_operator=equals&filter=Disease+Outbreaks

9. Caillaud D, Levréro F, Cristescu R, Gatti S, Dewas M, Douadi M, et al. Gorilla susceptibility to Ebola virus: the cost of sociality. Curr Biol. 2006;16:R489–91. PubMed https://doi.org/10.1016/j.cub.2006.06.017

10. Leroy EM, Epelboin A, Mondonge V, Pourrut X, Gonzalez JP, Muyembe-Tamfum JJ, et al. Human Ebola outbreak resulting from direct exposure to fruit bats in Luebo, Democratic Republic of Congo, 2007. Vector Borne Zoonotic Dis. 2009;9:723–8. PubMed https://doi.org/10.1089/vbz.2008.0167

11. Pigott DM, Golding N, Mylne A, Huang Z, Henry AJ, Weiss DJ, et al. Mapping the zoonotic niche of Ebola virus disease in Africa. Elife. 2014;3:e04395. PubMed https://doi.org/10.7554/eLife.04395

12. Shoemaker T, MacNeil A, Balinandi S, Campbell S, Wamala JF, McMullan LK, et al. Reemerging Sudan Ebola virus disease in Uganda, 2011. Emerg Infect Dis. 2012;18:1480–3. PubMed https://doi.org/10.3201/eid1809.111536

13. Grard G, Biek R, Tamfum J-J, Fair J, Wolfe N, Formenty P, et al. Emergence of divergent Zaire ebola virus strains in Democratic Republic of the Congo in 2007 and 2008. J Infect Dis. 2011;204:S776–84. PubMed https://doi.org/10.1093/infdis/jir364

14. Muyembe-Tamfum JJ, Mulangu S, Masumu J, Kayembe JM, Kemp A, Paweska JT. Ebola virus outbreaks in Africa: past and present. Onderstepoort J Vet Res. 2012;79:451. PubMed https://doi.org/10.4102/ojvr.v79i2.451

15. Maganga GD, Kapetshi J, Berthet N, Kebela Ilunga B, Kabange F, Mbala Kingebeni P, et al. Ebola virus disease in the Democratic Republic of Congo. N Engl J Med. 2014;371:2083–91. https://doi.org/10.1056/NEJMoa1411099

16. Nsio J, Kapetshi J, Makiala S, Raymond F, Tshapenda G, Boucher N, et al. 2017 Outbreak of Ebola virus disease in northern Democratic Republic of Congo. J Infect Dis. 2020;221:701–6. PubMed https://doi.org/10.1093/infdis/jiz107

17. ReliefWeb Perma.cc Records. Democratic Republic of Congo (DRC): Ebola outbreak emergency plan of action (EPoA) DREF Operation 1 [cited 2022 Jun 6]. https://perma.cc/PG7D-PEGQ

18. Lacroix A, Mbala Kingebeni P, Ndimbo Kumugo SP, Lempu G, Butel C, Serrano L, et al. Investigating the circulation of Ebola viruses in bats during the Ebola virus disease outbreaks in the Equateur and North Kivu Provinces of the Democratic Republic of Congo from 2018. Pathogens. 2021;10:557. PubMed https://doi.org/10.3390/pathogens10050557

19. Fick SE, Hijmans RJ. WorldClim 2: new 1-km spatial resolution climate surfaces for global land areas. Int J Climatology. 2017;37:4302–15. https://doi.org/10.1002/joc.5086

20. Hansen MC, Potapov PV, Moore R, Hancher M, Turubanova SA, Tyukavina A, et al. High-resolution global maps of 21st-century forest cover change. Science. 2013;342:850–3. PubMed https://doi.org/10.1126/science

21. Riitters K, Wickham J, O'Neill R, Jones B, Smith E. Global-scale patterns of forest fragmentation. Conservation Ecology. 2000;4:1–23. http://www.jstor.org/stable/26271763

22. Wan Z, Hook S, Hulley G. MOD11C3 MODIS/Terra Land Surface Temperature/Emissivity Monthly L3 Global 0.05Deg CMG V006 [Data set] [cited 2022 Jun 17]. NASA EOSDIS Land Processes Distributed Active Archive Center. https://doi.org/10.5067/MODIS/MOD11C3.006

23. Trabucco A, Zomer R. Global aridity index and potential evapotranspiration (ET_0) climate database version 3, 2022. https://doi.org/10.6084/m9.figshare.7504448.v4

24. Sims K, Reith A, Bright E, Kaufman J, Pyle J, Epting J, et al. LandScan Global 2022 data set. Oak Ridge National Laboratory. https://doi.org/10.48690/1529167

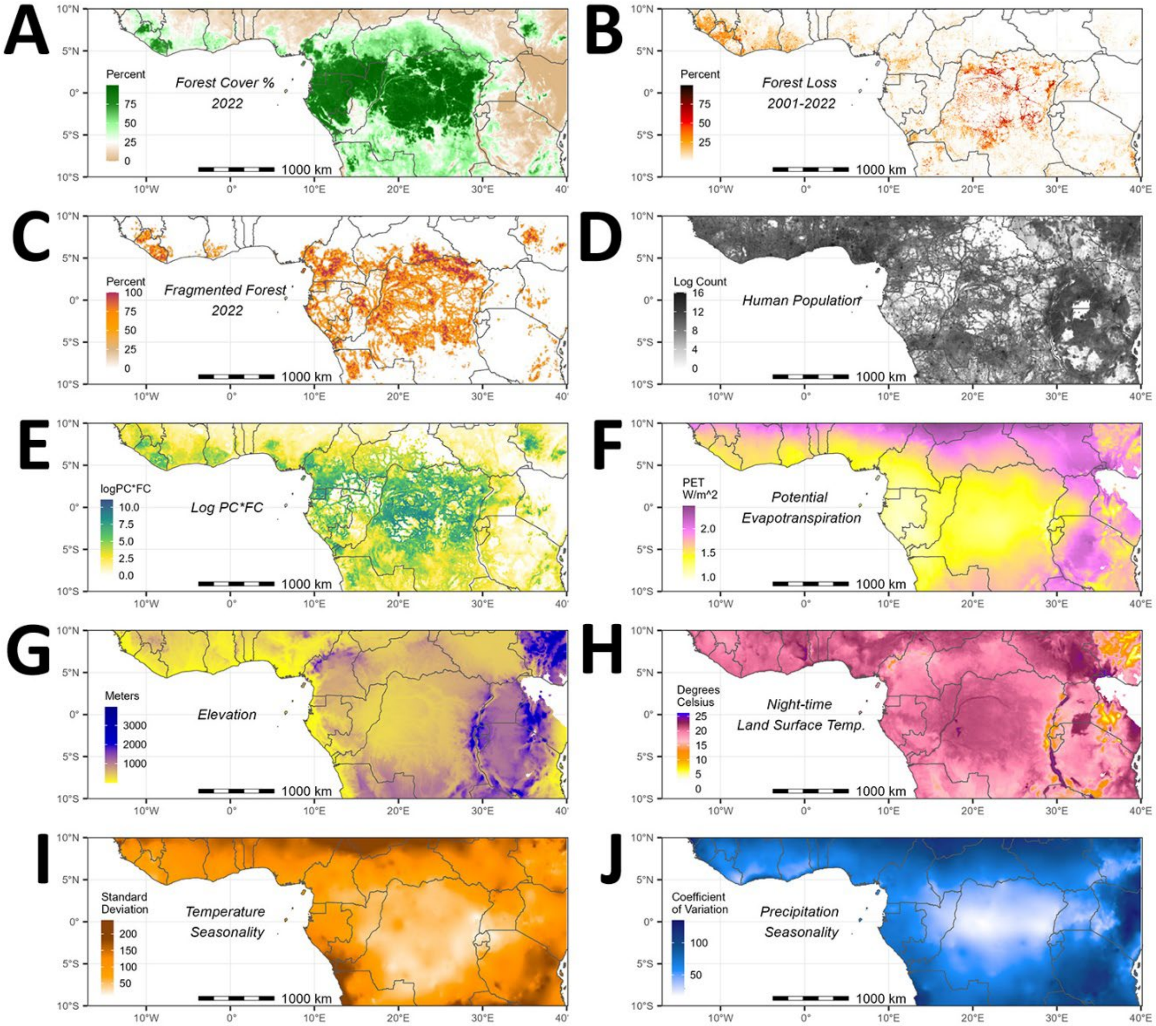

Appendix Figure 1. Maps displaying temporospatial data used in a predictive model for estimating annual *Ebolavirus* spillover potential.

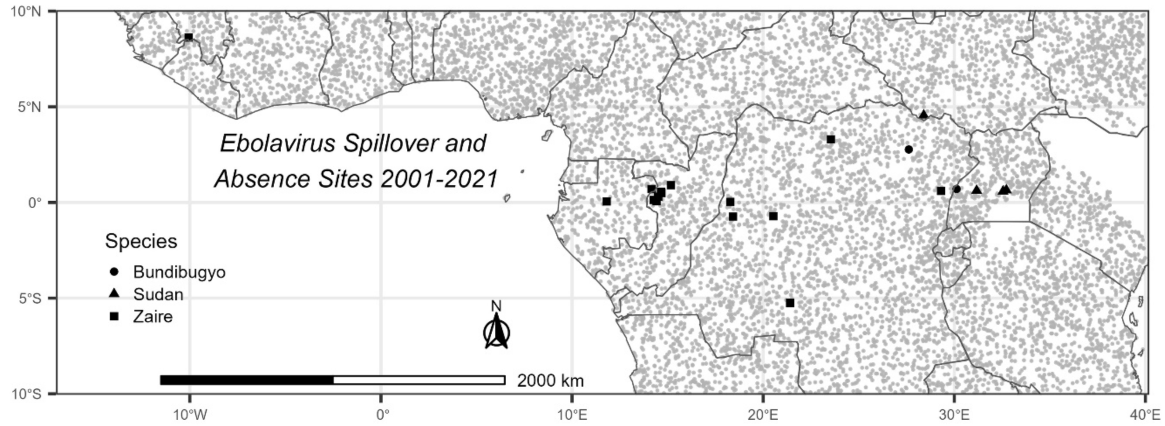

Appendix Figure 2. Map of *Ebolavirus* spillover sites, 2001–2021. Symbols indicate *Ebolavirus* species. Each gray dot represents a randomly generated location-year where spillover was not identified from 2001-2021.

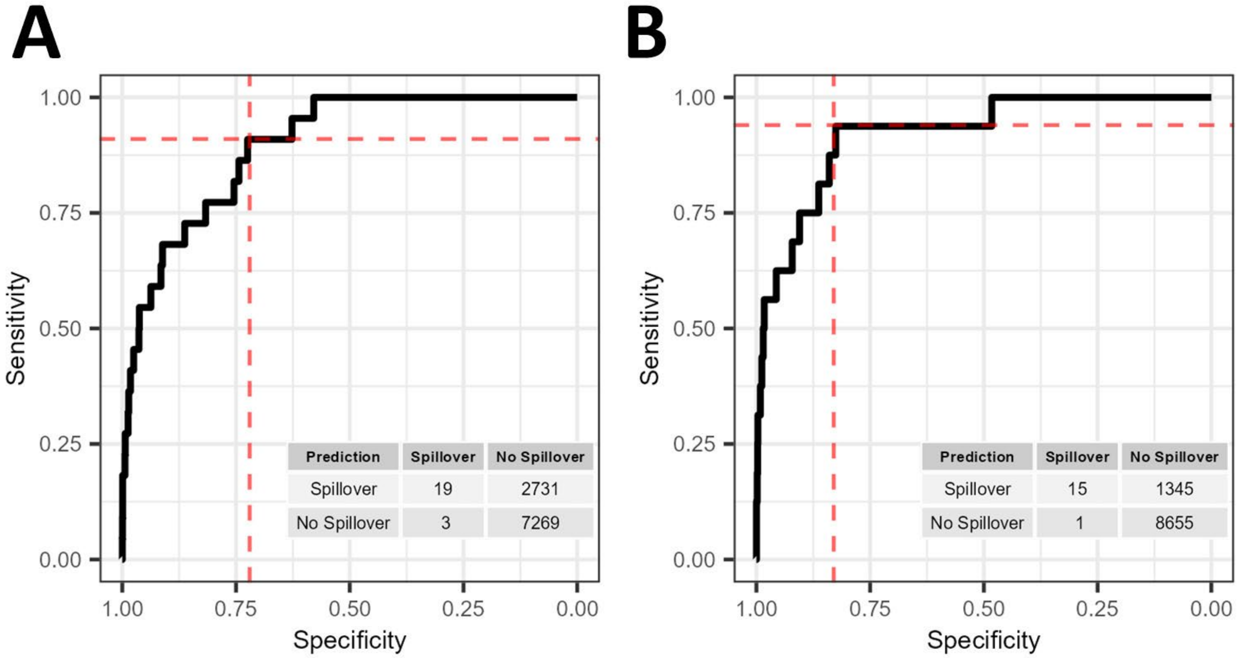

Appendix Figure 3. Receiver operator curves (ROC) visualizing sensitivity and specificity in predicting ebolavirus spillover from the All-species full and reduced analyses (A) and ZEBOV-only full and reduced analyses (B) based on leave-one-year-out cross validation and corresponding confusion matrices resulting from each analysis.

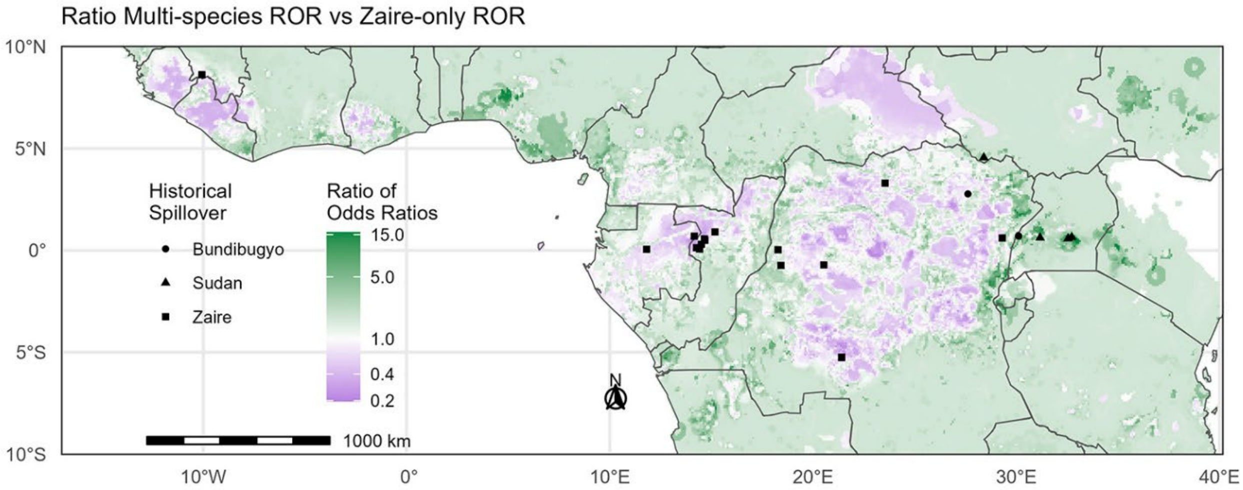

Appendix Figure 4. Map representing the ratio between estimated relative spillover odds in 2022 resulting from the multispecies relative to the ZEBOV-only analysis. Values >1 represent locations where relative spillover odds estimates from the Multi-species analysis were higher, while values <1 represent locations where estimates from the ZEBOV-only analysis were higher. ZEBOV, *Ebolavirus zaire*.

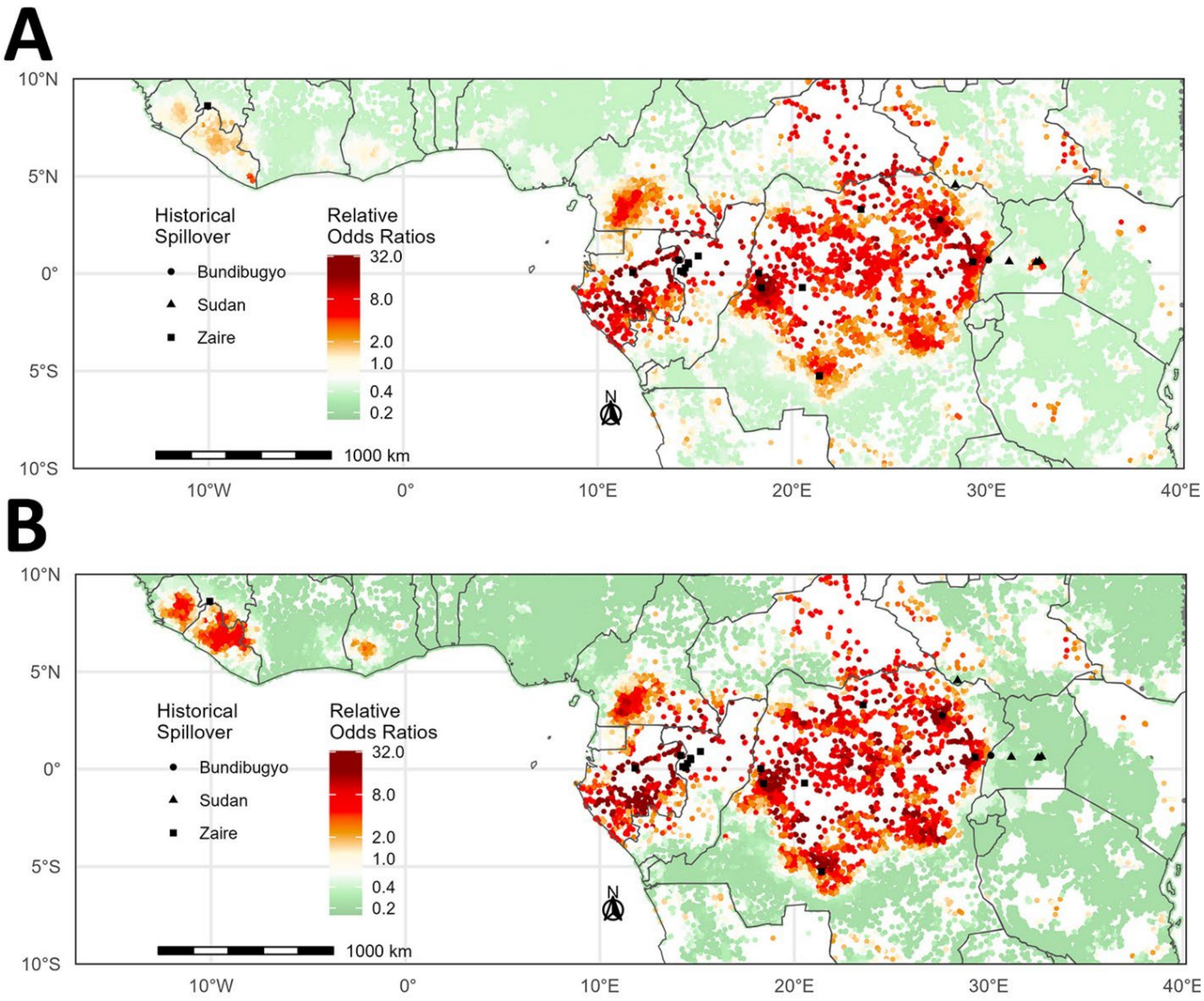

Appendix Figure 5. Relative spillover odds ratio estimates in 2022 resulting from sensitivity analysis which used health center coordinates throughout equatorial Africa as controls to train model of ebolavirus spillover. There were 57,920 heath facilities used as controls and at which estimates of relative spillover odds were made. Each dot in the figure represents a health facility. A) Multispecies model sensitivity; B) *Ebolavirus zaire* only model.

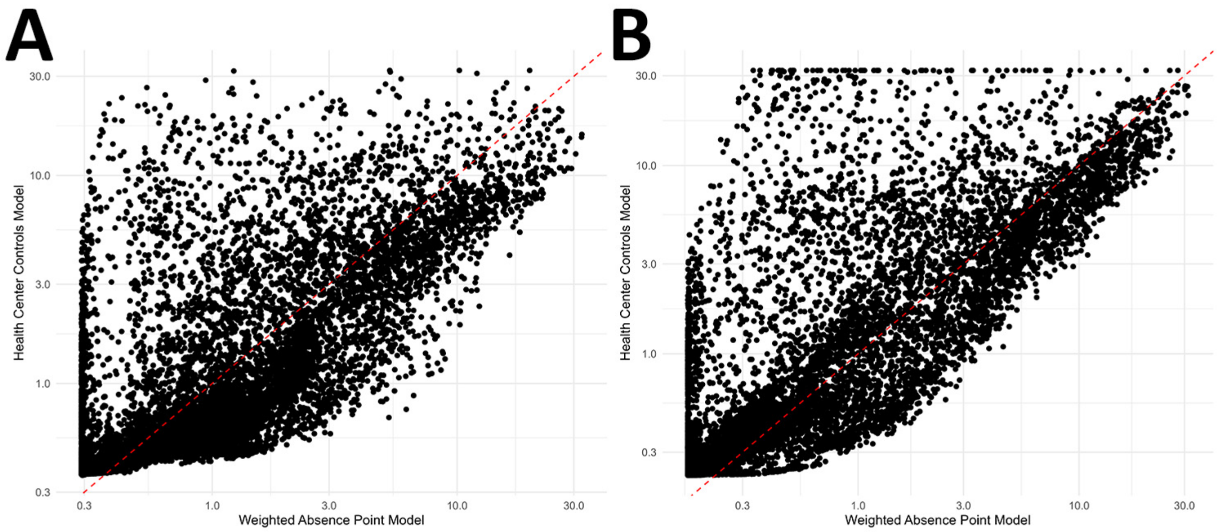

Appendix Figure 6. Estimated spillover relative odds ratios at the locations of health facilities resulting from the primary model trained on random absence points throughout the study area compared to a model whose controls were health facilities for the multispecies (A) and ZEBOV-only (B) analyses. ZEBOV, *Ebolavirus zaire*.

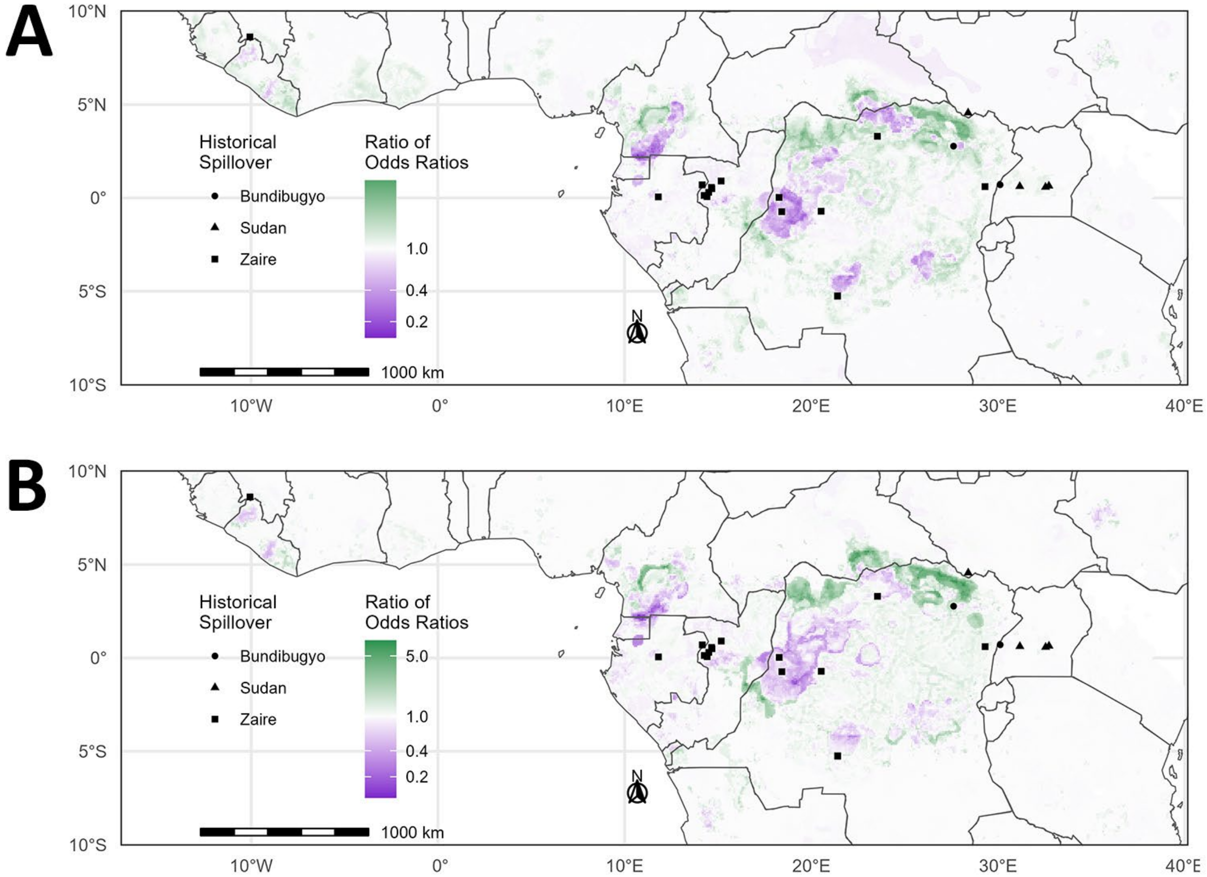

Appendix Figure 7. Sensitivity analysis of different forest definition cutoff values used to classify fragmented forests and their impact on relative spillover odds estimates. A) Multispecies model; B) ZEBOV-only model. Based on previous methods (*21*), fragmented forests are determined based on a binary classification of forests. Maps corresponding to each analysis represent the ratio between the estimated ratio of odds ratios (RORs) using the 80% forest cover as a cutoff versus 70% forest cover as a cutoff to define forests. Purple locations are those whose ROR estimates were higher when forest were defined as >80% forest cover. Green areas are those whose ROR estimates were higher when forests were defined as >70% forest cover. ZEBOV, *Ebolavirus zaire*.

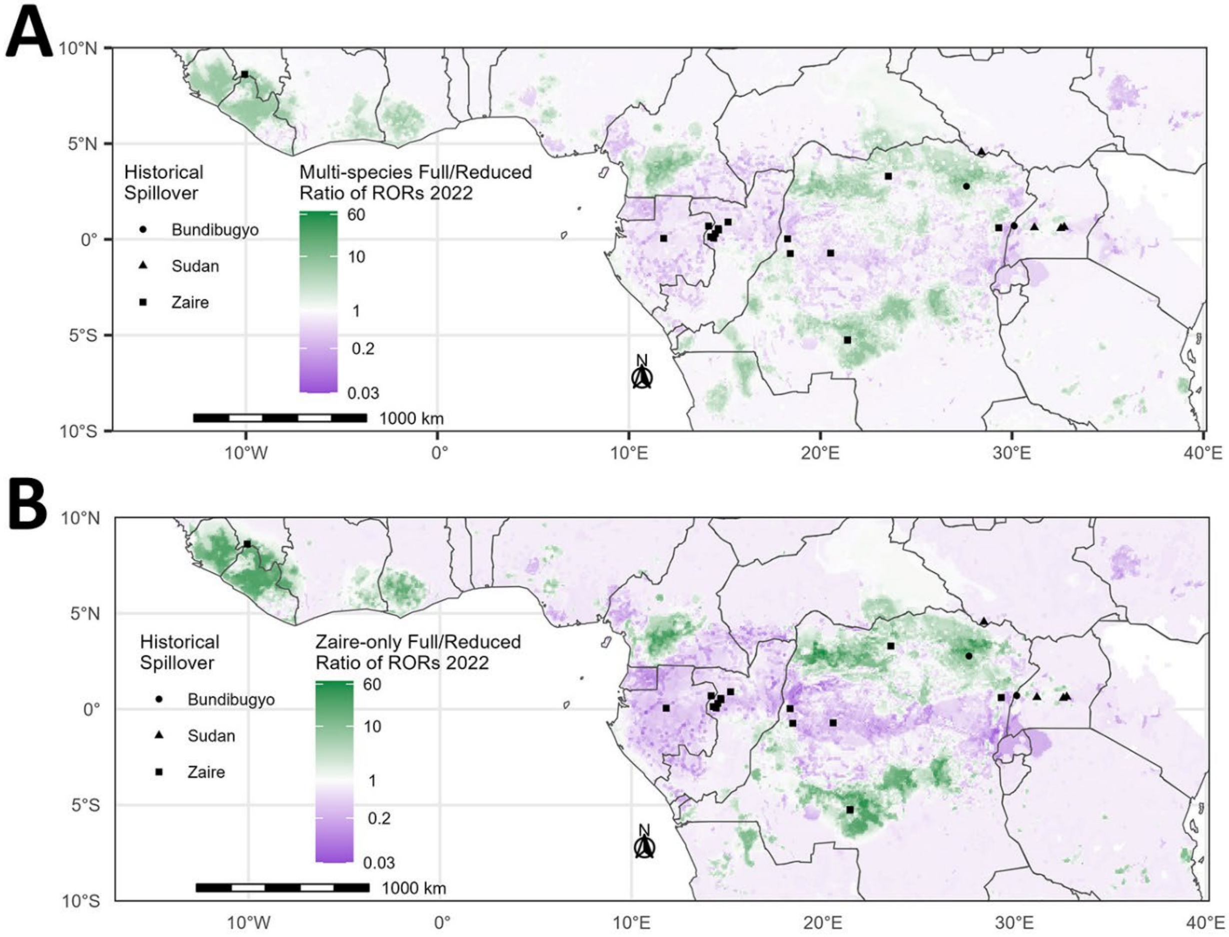

Appendix Figure 8. Ratio between estimated relative spillover odds in 2022 resulting from full models in the multispecies (A) and Zaire-only (B) analyses, relative to the reduced versions of the models that did not make predictions with covariates related to forest changes.
